# Shorter operative times following robotic-assisted transabdominal preperitoneal inguinal hernia repair (TAPP) compared to laparoscopic TAPP: the Danish Inguinal Randomized Controlled Trial (DIRECT)

**DOI:** 10.1007/s10029-025-03402-y

**Published:** 2025-07-09

**Authors:** Dinan Arunthavanathan, Rockson Liu, Ihsan Inan, Mehmet Oztoprak, Michael Festersen Nielsen

**Affiliations:** 1 Department of Surgery, Horsens General Hospital, Horsens, Denmark; 2 First Surgical Consultants, Oakland, CA USA; 3 Department of Surgery, Clinique Generale-Beaulieu, Geneva, Switzerland; 4 Department of Surgery, University Hospital of Southern Denmark, Aabenraa, Denmark

**Keywords:** Hernia, Inguinal hernia repair, Robotic-assisted inguinal hernia repair, Laparoscopic inguinal hernia repair

## Abstract

**Background:**

Despite the perception of higher procedural costs and longer operative time, robotic-assisted inguinal hernia repair has emerged as an alternative to the laparoscopic procedure. The present study was conducted to determine the time required for robotic and laparoscopic transabdominal preperitoneal (TAPP) inguinal hernia repair and to determine whether these time profiles differ between the two groups.

**Method:**

One hundred thirty-eight patients were randomized to a robotic-assisted r-TAPP (*n* = 74; 54%) or a laparoscopic l-TAPP (*n* = 64; 46%) procedure by experienced surgeons. The hernia defect was classified as either simple or complicated according to hernia size, involvement of the scrotum, and whether the hernia was a primary defect, a recurrence, or a bilateral defect.

**Results:**

Time from intubation to skin closure (*P* < 0.05) and from air insufflation to removal of instruments (*P* < 0.05) were shorter for the r-TAPP than for the l-TAPP procedure. This difference was observed for both simple and complex hernias, the difference between groups being larger for the complicated than for the simple defects. The analysis demonstrated that an additional 5 min were needed to dock the robotic platform and place the instruments. Despite this delay, the time required for the procedure remained shorter for the r-TAPP than for the l-TAPP repair.

**Conclusion:**

Robotic-assisted inguinal hernia repair is associated with a shorter operative time than conventional laparoscopy. While the time required for docking and instrument placement caused a minor delay of the procedure, the operating time for the robotic repair was shorter than for the laparoscopic procedure.

## Introduction

Since the first robotic-assisted transabdominal preperitoneal (r-TAPP) procedure for inguinal hernia was performed in 2007 [1], the number of robotic surgeries worldwide has increased. Many surgeons see little clinical benefit of r-TAPP over l-TAPP and consider these procedures only appropriate as training exercises for surgeons acquiring the fundamental skills needed for more complex robotic surgeries.

The introduction of robotic-assisted techniques in hernia surgery aims to overcome the limitations of conventional laparoscopy. These advantages include an enhanced auto-focus 3-dimensional optical view, more versatile articulated instruments mimicking the ability of the surgeon’s hands, and electromechanical systems that improve microsurgical precision. These innovations are intended to enhance the surgical quality of minimally invasive surgery and are expected to reduce the risk of complications compared to conventional laparoscopy.

Several studies have investigated the purported benefits of robotic-assisted inguinal hernia repair. A systematic review published by Qbbani et al., [2], which analyzed 19 studies involving 8,987 patients, found that robotic inguinal hernia repair was associated with longer operative times but fewer complications. The review revealed no significant differences in postoperative complications, pain, or hernia recurrence rates when compared to laparoscopic TAPP. Similar outcomes have been reported by Salani et al. [3], Li et al. [4] Miller et al. [5], and Prabhu et al. [6]. These studies have consistently demonstrated longer operative times and higher procedural costs associated with robotic inguinal hernia surgery. In contrast, we have recently published data that question these outcome results. In a retrospective analysis, Kaplan-Meier curves were constructed depicting the cumulative recurrence rate as a function of the observation time after surgery. A Cox proportional regression analysis performed based in the hazard ratio calculation demonstrated that the r-TAPP procedure was associated with a lower recurrence rate than l-TAPP [7]. Subsequently, data from a recent randomized controlled trial (RCT) indicate that the surgical stress response, as measured by postoperative CRP and IL-6 levels, was lower for r-TAPP than for l-TAPP [8]. Overall, these results suggest that the robotic TAPP procedure is associated with a lower surgical stress response, likely caused by the enhanced respect for tissue handling provided by the optimized ecosystem of the robotic platform. These features may explain the reported lower risk of recurrence. A particular feature provided by the robotic platform is an auto-focus 3-D imaging, permitting seamless zooming opportunity, allowing the surgeon to always be optimally close to tissues, which is expected to result in a less traumatic and faster surgical procedures. The present study was designed to address this question by conducting an analysis of the operative time required for the r-TAPP procedure and comparing those to the outcomes observed for the laparoscopic approach. To consider the additional operative steps only associated with robotic-assisted surgery, the repair procedure was divided into separate steps reflecting the different aspects of the operation. These time profiles were analyzed and compared between the robotic and the laparoscopic approach.

## Methods

### Study design

The study was conducted as a single-center randomized controlled trial (RCT) comparing the operative time for the l-TAPP to the r-TAPP procedure for inguinal hernia repair performed at the Department of Surgery, University Hospital of Southern Denmark, between November 2022 and May 2024. The study was conducted in accordance with the ethical standards of the Helsinki Declaration and was registered with ClinicalTrials.gov (NCT05839587) [9].

### Study subjects

Study subjects aged 18 or older referred for unilateral or bilateral inguinal hernia repair were considered eligible for the study. One hundred thirty-eight subjects were included and randomized for either a l-TAPP (*n* = 64; 46%) or a r-TAPP (*n* = 74; 54%) procedure. In the laparoscopic group, 38 (28%) patients were operated for a simple hernia, 26 (19%) for a complicated hernia. In the robotic group, 43 (31%) patients were operated for a simple hernia, 31(22%) for a complicated hernia. Patient characteristics are depicted in Table 1. Patients referred for emergency surgery due to an irreducible or incarcerated inguinal hernia were excluded from the study. Furthermore, patients with chronic pelvic pain, previous or concomitant cancer, psychiatric or addictive disorders, and patients with co-existing inflammatory or immunological diseases were also excluded from the study. A written informed consent was obtained from all study subjects before enrolment in the study.

**Table 1:** Patient characteristics

Variable	Level	r-TAPP (*N* = 74)	l-TAPP (*N* = 64)	*P*-value
Age (years), median (IQR)		61.5 (19.75)	66 (15)	0.09
Sex male, N (%)		62 (84)	53 (82)	0.90
BMI, median (IQR)		25.6 (5.2)	25.9 (4.3)	0.87
Smoking, N (%)	Never	40 (54.1)	35 (53.9)	0.9997
	Former	17 (23.0)	15 (23.1)	
	Current	17 (23.0)	15 (23.1)	
Alcohol use, N (%)	Never	7 (9.5)	9 (13.9)	0.35
	Social	3 (4.1)	7 (10.8)	
	Less than 7/14 per week	61 (82.4)	47 (72.3)	
	More than 7/14 per week	3 (4.1)	2 (3.1)	
ASA physical status, N (%)	ASA I	6 (8.1)	4 (6.2)	0.41
	ASA II	57 (77.0)	45 (69.2)	
	ASA III	11 (14.9)	15 (23.1)	
	ASA IV	0 (0)	1 (1.54)	
CCI, median (IQR)		2 (2)	3 (2)	0.06
Comorbidity, N (%)	Arterial Hypertension	23 (31)	26 (400)	0.36
	Diabetes Mellitus	2 (3)	3 (5)	0.66
	Heart disease	15 (20)	20 (31)	0.22
	Lung disease	11 (15)	8 (12)	0.85
	Cerebrovascular disease	4 (5)	6 (9)	0.51
	Dyslipidemia	8 (11)	13 (20)	0.20
	BPH	2 (3)	3 (5)	0.66
	Other comorbidity	13 (18)	17 (18)	1
Previous open surgery, N (%)	Contralateral herniotomy	11 (14.87)	7 (10.77)	0.64
	Ventral herniotomy	3 (4.05)	2 (3.08)	1
	Appendectomy	6 (8.11)	5 (7.69)	1
	Gynecologic^a^	5 (6.76)	2 (3.08)	0.45
	Other	8 (10.81)	7 (10.78)	1
	Any procedure ^b^	26 (35.14)	21 (32.31)	0.86
Previous laparoscopic surgery, N (%)	Contraleteral herniotomy	4 (5.41)	2 (3.08)	0.68
	Appendectomy	2 (2.7)	2 (3.08)	-
	Gynecologic^a^	1 (1.35)	1 (1.54)	-
	Cholecystectomy	3 (4.06)	6 (9.23)	0.30
	Other ^c^	4 (5.41)	6 (9.23)	0.51
	Any procedure ^b^	13 (17.57)	15 (23.08)	0.55
Hernia side, N (%)	Left	24 (32.43)	25 (38.46)	0.34
	Right	34 (45.95)	32 (49.23)	
	Bilateral	16 (21.62)	8 (12.31)	
Inguinoscrotal (n,%)		10 (13.52)	13 (20)	0.42
Recurrent (n,%)	Total	7 (9.46)	7 (10.77)	1
	Previous repair (Open)	6 (8.11)	5 (7.69)	0.90
	Previous repair (MIS)	1 (1.35)	2 (3.08)	
Complex hernia (n,%)		31(41.89)	26 (40.0)	0.96
Hernia defect diameter (cm), median, (IQR)^e^		2 (1)	2.5 (1)	0.57
Hernia type, n %	Lateral	51 (57.3)	42 (59.16)	0.18
	Medial	35 (39.33)	22 (30.99)	
	Both	3 (3.37)	7 (9.86)	

### Randomization of the study subjects

Patients were randomly allocated to the r-TAPP or l-TAPP procedure using the randomization tool in Research Electronic Data Capture (REDCap). To ensure a random allocation, neither the project manager nor any member of the research or surgical team had access to the randomization code. Following recruitment, the project manager conducted the randomization in REDCap. During the surgical procedure the hernial defect was classified as a simple or complicated hernia. A simple hernia was defined as an inguinal protrusion with only a slight involvement of the inguinal canal. A complicated hernia was defined as a large defect with a hernial sac involving the inguinal canal, a bilateral or recurrent hernia. The allocation of each of the 138 procedures to the appropriate study group was made by the surgeon on the day of surgery.

### The surgical procedure

All procedures were performed by four consultant surgeons with extensive experience in robotic and laparoscopic inguinal hernia surgery. All surgeons were certified in robotic surgery and had performed at least 100 r-TAPP and l-TAPP procedures. Patients were randomly allocated to either r-TAPP or l-TAPP. The procedures were carried out in random order throughout the study period.

The surgical procedure was conducted according to a standardized protocol [10]. Minor intraoperative modifications, such as adjustments in mesh size and port placement, were permitted to accommodate anatomical variations while adhering to predefined surgical principles.

### r-TAPP repair

R-TAPP was performed using the da Vinci Xi^®^ system (Intuitive Surgical Inc., Sunnyvale CA, USA). The procedure was performed using a 3-point access with 8 mm trocars: a camera port at the umbilicus, and two additional working ports laterally to the rectus abdominis muscles on each side. A pneumoperitoneum was established using a Veress needle, abdominal pressure set to 15 mmHg followed by docking. During the procedure, the peritoneum was incised and the hernial content reduced as previously described. A 15 × 12 cm ProGrip™ Self-fixating mesh (Medtronic Inc., Minneapolis, MN, USA) was cut to size and placed to reinforce the myopectineal orifice. The peritoneum was entirely reinserted with barbed absorbable sutures [V-LOC™ (Medtronic Inc., Minneapolis, MN, USA)]. The robotic platform was undocked, exsufflation followed by port removal and skin closure using absorbable sutures.

### l-TAPP repair

L-TAPP was performed according to the principles as described above. Following positioning of the patient in the supine position, a 12-mm camera port was placed at the umbilicus, and two 5-mm working ports were inserted laterally to the rectus abdominis muscles. The rest of the procedure for the l-TAPP was identical to the r-TAPP.

### Administration and timing of the interventions

Both interventions were administered under general anesthesia, with patients fasting for at least 6 h before the procedure. No antibiotic prophylaxis or corticosteroids were administered. Pain management was standardized across both groups, with patients receiving a combination of systemic analgesics (paracetamol and opiates) and local anesthetic infiltration at the port sites (20 mL Bupivacaine 0.5%). This did not include the administration of Non-Steroid Anti-Inflammatory Drugs (NSAID).

### Time recordings during the surgical procedure

Intraoperative and postoperative outcomes were recorded using structured forms. During the surgical procedure, recordings were made regarding hernia type (direct, indirect or both), hernia defect size (measured at an insufflation pressure of 8 mmHg), mesh size, intraoperative complications, as well as surgical and anesthesia time. Time recordings during the surgical procedure were divided into 4 Steps. Step 1 consisted of the time from placement of the Veress needle until incision of the peritoneum. This recording involved port placement, docking of the robotic platform, and placement of the instruments into the abdominal cavity. Step 2 recorded the time from incision of the peritoneum until removal of the surgical instruments. Step 3 recorded the time from instrument removal to skin closure. During this period, the time for removing the robotic platform was included. Step 4 recorded the time from placement of the Veress needle until skin closure was completed. The operating time was calculated as the sum of Step 1 and 2.

### Effects of the time recordings on procedure efficiency

To compare the efficiency of the robotic to the laparoscopic repair, the operative time and an estimated turnover of 50 min between procedures were used to calculate the expected number of procedures that are feasible to conduct during working hours from 08.00 a.m. until 5 p.m.

### Statistical analyses

Descriptive statistics were used to summarize the baseline characteristics of the study population. Continuous variables were reported as medians with interquartile ranges (IQR) due to their non-normal distribution, while categorical variables were summarized as counts and percentages. Procedure times were calculated as means ± standard error of the mean (SEM). Comparative analyses were conducted between the l-TAPP and r-TAPP groups to assess potential differences in baseline characteristics. Statistical analyses were performed using Stata^®^ and R. Categorical variables were compared using the student t-test for non-paired analyses. A two-sided p-values were calculated to determine the statistical significance of the differences observed. A p-value of less than 0.05 was considered statistically significant. All statistical analyses were conducted using R version 4.4.0.

The primary outcome of the study was to determine the time required for conducting robotic-assisted and laparoscopic TAPP procedures for inguinal hernia repair. These recordings were analyzed and compared between groups. The calculation of this outcome was a secondary analysis of a dataset in which CRP was used to compare the surgical stress response associated with TAPP inguinal hernia repair. The sample size for this calculation required 135 study subjects, adjusted for a 10% dropout [8]. Based on the time recordings presented in the present study, a post hoc power analysis was conducted using a Monte Carlo simulation, assuming a difference of 15% between groups and a level of 5% to obtain statistical significance. This simulation demonstrated a power of 99% associated with the statistical comparison between the Steps 1 to 4 time recordings, demonstrating a risk of type 2 error of less than 1%.

## Results

### Patient demographics and baseline characteristics

One hundred thirty-eight patients referred for inguinal hernia repair were included in the study. Patients’ characteristics for study subjects randomized to either r-TAPP or l-TAPP are depicted in Table 1. The two groups consisted of 84 and 82% males. The median (IQR– interquartile range) BMI was 25.6 and 25.9 kg/m^2^. The percentage of smoking, alcohol intake, ASA score distribution, the presence of comorbidities among the study participants as well as the hernia characteristics, did not differ between the two groups.

The intraoperative findings are depicted in Table 2. A comparable need for adhesiolysis was recorded in the two study groups (16 vs. 17%; P = NS). No difference in conversion rate was observed. Noteworthy, a slightly higher blood loss was observed in the l-TAPP group.

**Table 2:** Intraoperative findings

Variable	r-TAPP (*N* = 74)	l-TAPP (*N* = 64)	*P*-value
Adhaesiolysis, n (%)	12 (16.22)	11 (16.92)	1
Fibrosis, n (%)	4 (5.41)	5 (7.69)	0.73
Blood loss in mL, median (IQR)	5 (10)	7.5 (15)	0.04
Conversion to open surgery, n (%)	0	1 (1.54)	-
Bowel injury	1	1	-
Hernia sack incised and not reduced	0	2	-
Mesh size (craniocaudalt) median, (IQR)	12 (1)	12 (0)	0.01
Mesh size (Mediolateral), median, (IQR)	15 (0)	15 (0)	0.01
Bilateral mesh, n	5	3	-

### Time analysis

#### Application of Veress needle to peritoneum incision (Step 1)

The time required for the set-up of the procedure did not differ for the simple or complex hernias for the l-TAPP (10 ± 1 vs. 11 ± 1 min; *P* = 0.39) and the r-TAPP (16 ± 1 vs. 16 ± 1 min; *P* = 0.70 procedures, respectively. However, the set-up time was 5 min longer for the robotic procedure than for the laparoscopic procedure (*P* < 0.01). This observation demonstrates that the docking procedure and the additional time required for placement of the instruments result in a 5-minute delay of the preoperative set-up procedure. Notably, this delay was reproducible and independent of the hernial defect.

#### Incision of the peritoneum to remove instruments (Step 2)

The time required for the step 2 procedures took longer for the simple compared to the complex repairs for both the l-TAPP (50 ± 2 vs. 81 ± 7 min; *P* < 0.01) and the r-TAPP (35 ± 2 vs. 57 ± 4 min; *P* < 0.01) procedures. Furthermore, the r-TAPP procedure was quicker than the l-TAPP repair for both the simple (35 ± 2 vs. 50 ± 2 min; *P* < 0.01) and complex (57 ± 4 vs. 81 ± 7 min; *P* < 0.01) repairs. The time gap between the two procedures was larger for the complex than for the simple hernias (24 vs. 15 min; *P* < 0.01).

#### Total operative time (application of Veress needle to removal of instruments)

**Fig. 1:**
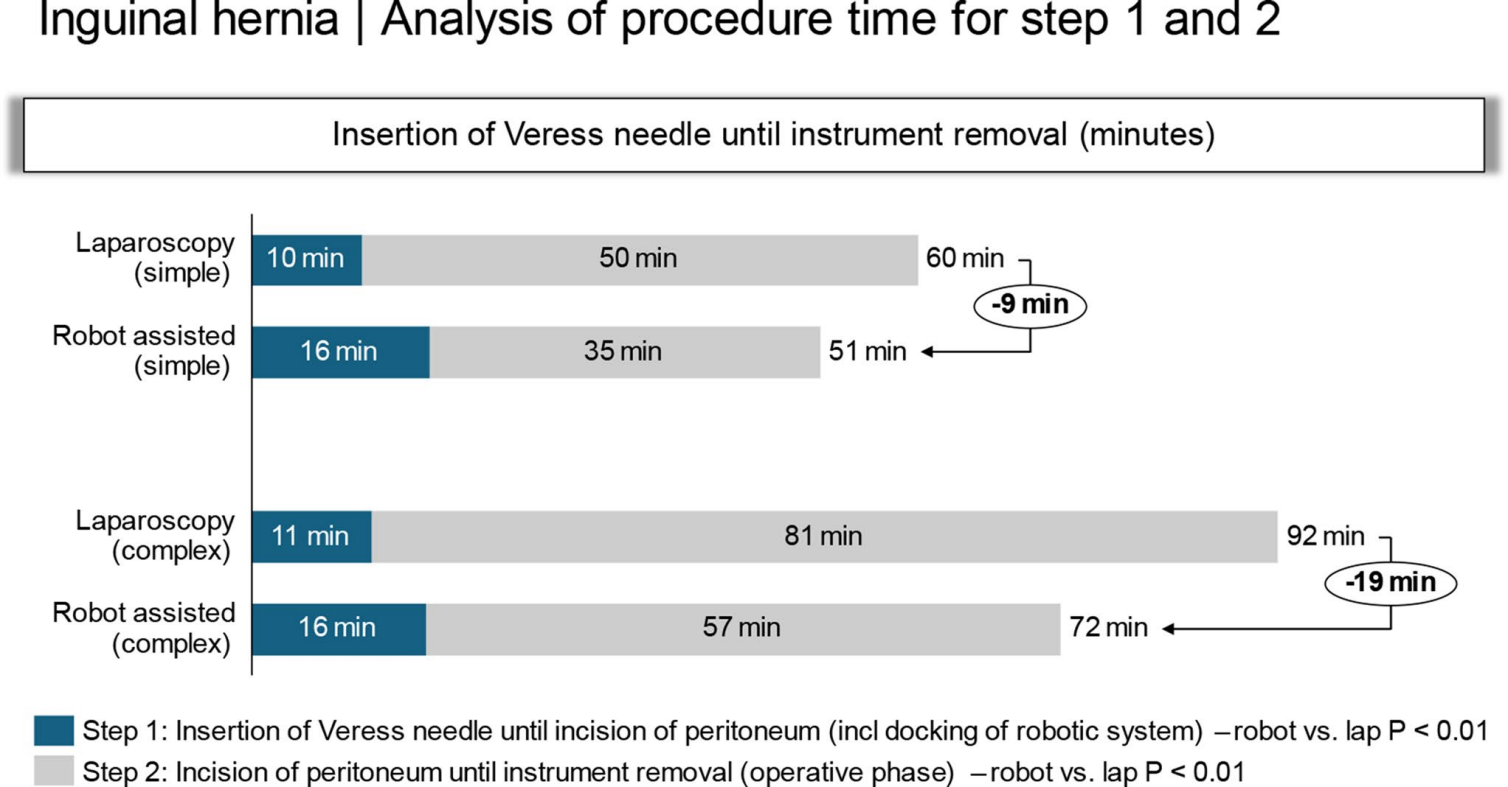
The operative times required for Steps 1 and 2 are depicted in minutes. The time differences between the l-TAPP and r-TAPP procedures for simple and complex hernias are shown

**Fig. 2:**
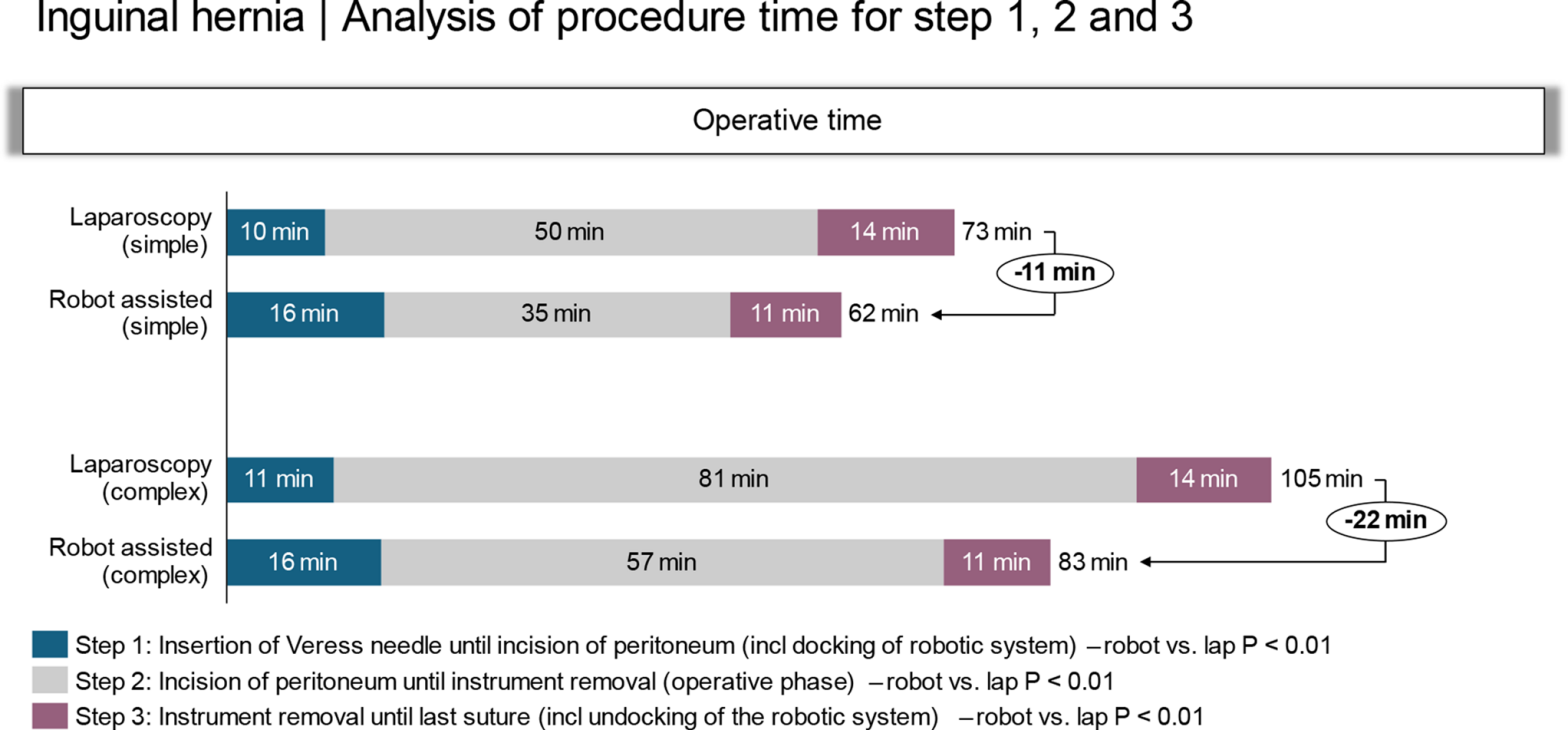
Time for Steps 1, 2 and 3 of the inguinal hernia procedure. The time required for each step is depicted in minutes

The operative time constitutes the time for Steps 1 and 2. Despite the 5 min additional time needed for the docking procedure and for placement of the instruments (step 1), the operative time was shorter for the r-TAPP than for the l-TAPP repair for both simple 51 ± 3 vs. 60 ± 3 min; *P* < 0.01) and complex hernias (72 ± 4 vs. 92 ± 7 min: *P* < 0.01) (Fig. 1). Moreover, the time required by the two procedures was greater for the complex than for the simple defects (Fig. 2).

#### Removal of the instruments to skin closure (Step 3)

The time required for removal of the instruments until skin closure was comparable for the simple and complex hernias for both the r-TAPP (11 ± 2 vs. 11 ± 1 min; *P* = 0.8) and l-TAPP (14 ± 1 vs. 14 ± 1 min; *P* = 0.9) procedures. The time for this part of the operation did not differ between the two surgical procedures (*P* = 0.13).

#### Application of Veress needle to skin closure (Step 4)

This time required from the application of Verres needle to skin closure was longer for the complex simple than for the simple repairs for both the laparoscopic (73 ± 3 vs. 105 ± 7 min; *P* < 0.01) and the robotic-assisted repair 62 ± 4 vs. 83 ± 4 min; *P* < 0.01). As demonstrated for the operative time, the robotic repair was faster than the laparoscopic repair for both simple 62 ± 4 vs. 73 ± 3 min; *P* < 0.01) and complex (83 ± 4 VS 105 ± 7 min; *p* < 0.01) hernias.

### Efficiency of the laparoscopic and robotic-assisted repair

The number of cases that may be carried out in one operating room block, assuming 540 min (9 h) available for the procedures, is depicted in Table 3. Based on the operative times for the l-TAPP and r-TAPP procedures and an estimated turnover time between procedures of 50 min, 4 simple l-TAPP and 5 simple R-TAPP procedures can be completed in one operating room block day. One extra hernia repair gained by performing the operation robotically increases the operative room utilization by 25%, compared to laparoscopy. Similarly, the time savings from robotic surgery in complex hernias allows for an additional procedure. Whereas only 3 complex hernias would be repaired laparoscopically in one block, 4 r-TAPP repairs could be completed, which would improve block utilization by 33%.

**Table 3:** Time profiles for patients with simple and complex inguinal hernias undergoing either a l-TAPP or a r-TAPP repair. Step 1: time from placement of the Veress needle to peritoneal incision. Step 2: time from peritoneal incision to removal of surgical instruments. Step 3: time from instrument removal to skin closure. Step 4: total time from Veress needle placement to skin closure

	l-TAPP		*r*-TAPP		
	Simple (*n* = 38)	Complicated (*n* = 26)	Simple (*n* = 43)	Complicated (*n* = 31)	*P*-value
Step 1	10 ± 1	11 ± 1	16 ± 1	16 ± 1	*P* < 0.01
Step 2	50 ± 2	81 ± 7	35 ± 2	57 ± 4	*P* < 0.01
Total op (Step 1 + 2)	60 ± 3	92 ± 7	51 ± 3	72 ± 4	*P* < 0.01
Step 3	14 ± 1	14 ± 1	11 ± 2	11 ± 1	*P* = 0.13
Step 4	73 ± 3	105 ± 7	62 ± 4	83 ± 4	*P* < 0.01

## Discussion

The present study demonstrates that r-TAPP inguinal hernia repair takes less time than l-TAPP repair. This difference was noted despite the additional 5 min docking and instrument setup time involved in the r-TAPP procedure (Step 1). Furthermore, the analysis demonstrates that the shorter operative time for the r-TAPP repair was due to less time required for dissection, mesh handling, and the peritoneal defect suturing. This finding was observed for the simple repairs (15 min shorter operative time), but was particularly evident for the complex cases i.e., patients with recurrent hernias, large inguinoscrotal sacs, and bilateral hernias (24 min shorter operative time).

To our knowledge, this is the first study to demonstrate that r-TAPP is faster than l-TAPP for inguinal hernia repair. This conclusion stands in sharp contrast to the only RCT, and several meta-analyses and clinical trials published to date. A review conducted in 2021 by Qabbani et al. based on 19 studies and a total of 8987 patients demonstrated that robotic-assisted inguinal hernia repair was associated with longer operative times, lower admission rates, and fewer complications [2]. These outcomes were subsequently confirmed by Solani et al. in a review comprised of 9 articles with a total of 7589 patients [3]. In this review, laparoscopic and robotic hernia repairs were associated with similar safety parameters and postoperative outcomes, but longer operative times for patients undergoing a robotic procedure. Similar outcomes have been reported by Khewater [11] and Huerta et al. [12]. In the multicenter, single-blinded, prospective randomized RIVAL trial [13], 102 patients were randomized to either a laparoscopic or a robotic repair. Compared with the traditional laparoscopic repair, the robotic TAPP was associated with longer operative times, higher procedural costs, and no added ergonomic benefits to surgeons.

Several factors may account for the discrepancy between these studies and the outcome of this study.

First, the present study was designed as a randomized controlled clinical trial (RCT) based on a large cohort of participants. The shorter operative time reported for robotic repair may reflect a more comprehensive and robust statistical analysis than can be obtained in a meta-analysis.

Second, to achieve a detailed analysis of the time required for the repair, the operation was divided into 4 steps. This study design was chosen to time the various technical and procedural challenges encountered during the TAPP repair. In particular, emphasis was placed on taking into account the impact of the docking procedure, the time required to introduce the robotic instruments into the abdominal cavity, and the potential delay caused by removing the robotic platform after the operation. This novel approach provides a robust time profile analysis that allows for a head-to-head comparison between robotic and laparoscopic repairs.

Third, all robotic and laparoscopic repairs were performed by experienced hernia surgeons. At the time of enrollment, all surgeons had each performed over 200 r-TAPP and 200 l-TAPP procedures. This level of expertise may not have been matched in the meta-analyses, potentially introducing a bias. Additionally, a significant variation of the surgical skills among surgeons may have introduced heterogeneity between groups, potentially influencing the outcomes of the statistical analyses.

Fourth, prior studies included surgeries performed with prior generation robotic platforms that were less sophisticated and less easy to use than the daVinci Xi platform. In our study, the Xi was used in all procedures. The benefit of the robotic platform lies in the precision and dexterity of the robotic instruments, the auto-focus enhanced three-dimensional image allowing close to the tissue dissection and tremor filtration. These features are enhanced with each generation of the robot. Moreover, the daVinci Xi introduced enhancements that reduce docking times. Since these new features and enhancements together can reduce setup time and speed up the conduct of the repair, the inclusion of multiple generations of robots in a single study can introduce a source of error that may have obscured a time difference between the robotic and the laparoscopic repairs.

Fifth, our study was conducted at a single center. A multi-center study would likely increase the power of the study. However, when taking the prevailing statistical differences into account, we consider this error to be small with no significant impact on the study outcome.

One of the reported reasons robotic surgeries take longer than lap is the complex and time-consuming setup process [3, 5, 6, 11, 14, 15]. For inguinal hernia repair, it has been argued that this delay undermines the technical benefits of robotic surgery to the point where it may be deemed ineffective and unsuitable for groin hernia surgery [14, 16–18]. The present study challenges this perspective. Our findings show that while the docking and alignment of the robotic instruments are added steps, these only extended the setup time by 5 min (Step 1). However, this added time was offset by a faster surgical execution (Step 2), resulting in an overall reduction in operative time by 11 min for simple hernias and 22 min for complicated defects (Step 4, Table 3).

**Fig. 3:**
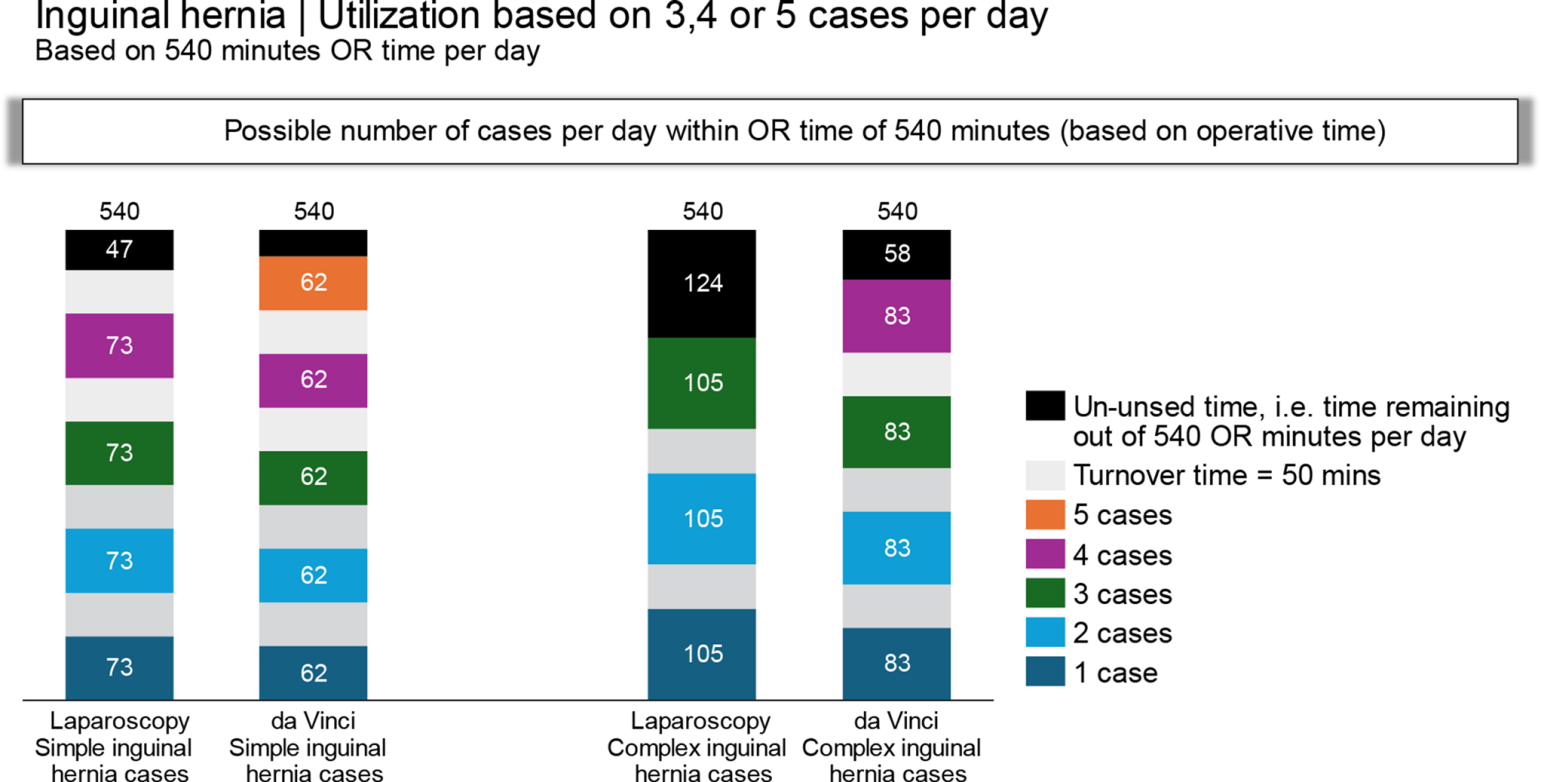
Estimated number of simple and complex hernia procedures during a 540-minute (9-hour) shift. The calculation is based on the duration of Step 4 of the repair procedure (Table 3) and an estimated turnover time of 50 min between procedures

In the context of increasing the efficiency of the robotic repair, the time profiles outlined for Steps 1 to 4 should be reviewed. These steps cover the time needed for docking and removal of the robotic platform after the operation. Likely, a critical review of these steps holds the key for improving the efficiency of robotic repair. The impact of these time profiles on the efficiency of the robotic procedure is depicted in Table 3 and in Fig. 3, where the impact on the number of procedures that can be performed on a single day demonstrates the importance of reducing the time needed for set up and removal of the instruments as well as to focus on reducing the turnover time between the procedures. An important tool to achieve this goal is to establish a team comprised of the surgeon, the nurses, the OR personnel, and the anesthesiologist that collaborates on reducing the time required for preoperative and postoperative procedures. Particularly, a close collaboration between the surgeon and the anesthesiologist is important to ensure the proper time for exturbation and a time-efficient workup for the next procedure. In this context, there is room for improvement; however, the benefits appear to be worthwhile, both from a financial point of view and in the context of ensuring the best quality of care, which serves as a significant motivational factor for implementing the robotic-assisted procedure in inguinal hernia surgery. While the present study addresses the impact of robotic-assisted inguinal hernia repair on time efficiency, the impact of recommending this approach on healthcare costs remains an important factor to consider that ought to be assessed in future analyses.

In conclusion, we demonstrated that the total time required for the surgical dissection, mesh handling, and repair of the peritoneal defect was significantly shorter for r-TAPP than for l-TAPP (Step 2). While this difference was demonstrated for simple hernias (15 min), it was particularly evident for the repair of complicated defects (24 min). These time savings significantly offset the additional 5 min needed for the robotic docking and instrument placement. Taking into account that robotic surgery has been linked to a lower surgical stress response [8] and reduced risk of recurrence [7], the results presented in this study provide further evidence to support the significant clinical benefits of using the robotic surgical platform in TAPP inguinal hernia repairs.
